# Studies on the Effect of Lipofectamine and Cell-Penetrating Peptide on the Properties of 10-23 DNAzyme

**DOI:** 10.3390/molecules28093942

**Published:** 2023-05-07

**Authors:** Huanhuan Liu, Yang Li, Shanshan Du, Chenhong Wang, Yuexiang Li, Ruiyuan Cao, Weiguo Shi, Shihui Liu, Junlin He

**Affiliations:** 1 School of Pharmaceutical Sciences, Guizhou University, Guiyang 550025, China; huanhuanliu2023@163.com; 2 State Key Laboratory of Toxicology and Medical Countermeasurements, Beijing Institute of Pharmacology and Toxicology, Taiping 27, Beijing 100850, China; liyang920312@163.com (Y.L.); shanshandu1992@163.com (S.D.); wang_chenhong@126.com (C.W.); lyx1986528@126.com (Y.L.); 21cc@163.com (R.C.); shiweiguo@bmi.ac.cn (W.S.)

**Keywords:** 10-23 DNAzyme, cell-penetrating peptide, lipofectamine 2000, observed rate constant, T_m_, CD spetra, PAGE analysis

## Abstract

Cationic polymeric materials and cell-penetrating peptides (CPPs) were often used as the delivery vectors in the evaluation of nucleic acid therapeutics. 10-23 DNAzyme is a kind of potential antisense therapeutics by catalytic cleavage of the disease-related RNAs. Here, lipofectamine 2000 and Tat peptide were evaluated for their effect on the catalytic activity of 10-23 DNAzyme, with the observed rate constant, thermal stability, CD spectra, and PAGE analysis, with a duplex DNA mimicking DNAzyme-substrate as a control. It was shown that the cationic carriers had a negative effect on the catalytic performance of the 10-23 DNAzyme. Significantly, the destabilizing effect of the cationic carriers on the duplex formation was noteworthy, as a duplex formation is an essential prerequisite in the silencing mechanisms of antisense and RNAi.

## 1. Introduction

Nucleic acid therapeutics have gained great improvements for drug-like properties, including enhanced nuclease stability and target-binding affinity, less toxicity, and other favorable pharmacokinetic properties, with oligonucleotide medicinal chemistry [1,2,3]. However, efficient delivery is still a great challenge for therapeutic oligonucleotides to reach the extra-liver organs or tissues by systematic administration [4,5]. Numerous natural and synthetic polymers have been exploited as transmembrane delivery facilitators through nanoparticle encapsulation or covalent conjugation of therapeutic oligonucleotides [6,7]. Among them, the positively charged LNPs and copolymers and cell-penetrating peptides (CPPs) have been used for short oligonucleotides and mRNA in bench and clinical studies [8,9,10,11].

**Figure 1:**
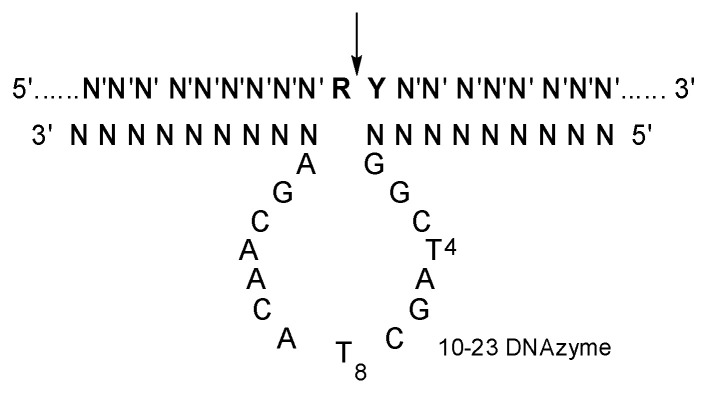
The secondary structure of 10-23 DNAzyme-substrate complex. N constitutes the two recognition arms, with base-pairing specificity for N′ of the substrate. R is purine and Y is pyrimidine residue in the substrate, and the cleavage site indicated by an arrow.

Moreover, 10-23 DNAzyme is an artificially selected catalytic DNA molecule with the capability of cleaving complementary RNAs (Figure 1) [12]. It has been extensively explored as a kind of potential therapeutics against various diseases with mutated RNAs (mRNAs and miRNAs et al.), with high specificity and little immunogenicity [13,14,15,16,17,18]. Various delivery materials have been used in the preliminary evaluation of 10-23 DNAzyme [19,20]. Obviously, the catalytic activity of 10-23 DNAzyme was significantly affected by the delivery materials. Several DNAzymes, including Dz13, behaved like an antisense oligonucleotide in cells when transfected with FuGENE6 [21]. On the other hand, a 10-23 DNAzyme was effective against TNF-α in vivo by gold nanoparticle delivery, but it was inactive when delivered with a positively charged lipofectamine carrier [22]. A cationic comb-type copolymer PLL-g-Dex was developed for 10-23 DNAzyme delivery [23]; it had no influence on the cleavage reaction under single-turnover conditions but accelerated the turnover step under multiple turnover conditions [24,25,26,27,28].

CPPs and lipofectamines are the most often used delivery materials in the preliminary evaluation of 10-23 DNAzyme and other oligonucleotide therapeutics. As their sidechain functional groups (amino, guanidinyl, and amido groups) [29,30] are capable of forming hydrogen bonding and electrostatic interactions with the residues in the catalytic loop and recognition arms of 10-23 DNAzyme, a detailed analysis of the effect of Tat [31] and lipofectamine 2000 on the catalytic activity and DNAzyme-substrate complex formation were studied. Our research demonstrated that these cationic polymers have a critical effect on the properties of 10-23 DNAzyme. Significantly, the duplex formation was also negatively affected, which is a prerequisite for substrate binding and the right folding of an active 10-23 DNAzyme [32], as well as the silencing ability of siRNAs and ASOs.

## 2. Results and Discussion

10-23 DNAzyme (DZ01: [5′-d(TGC TCT CCA GGC TAG CTA CAA CGA CCT GCA CCT)-3′] was designed against a DNA-RNA-DNA chimeric substrate D19R [5′-d(AGG TGC AGG)-AU-d(GG AGA GCA)-3′] [19] with cleavage reactions measured in the presence of 2 mM Mg^2+^. Registered peptide GRKKRRQRRR (Tat) and lipofectamine 2000 were used to form complexes with DZ01 with different charge ratios, respectively. The encapsulated DZ01 was used for all the evaluations. The full-DNA substrate D19 [5′-d(AGG TGC AGG ATG GAG AGC A)-3′] was used to bind DZ01 for the CD spectra and thermal stability measurement. In addition, as the duplex formation between the recognition arms of 10-23 DNAzyme and the substrate was a critical step for the active conformation and the catalytic reaction, the two recognition arms were combined with being as D19S [5′-d(TGC TCT CCA TCC TGC ACC T)-3′], and it was fully complementary to the substrate D19. This DNA duplex D19S-D19 in the same encapsulation complexes was used as a comparison.

### 2.1. Catalytic Reactions of 10-23 DNAzyme Encapsulated with the Cationic Polymers

Single-turnover conditions were used to study the effect of cationic polymers on the cleavage reaction of 10-23 DNAzyme [33], by which only the catalytic reaction step was investigated for the effect of Tat or lipofectamine. As shown in Table 1, the cleavage reaction of DZ01 was negatively affected when packaged in the polymer complex, with a dependence on the charge ratios. When the total positive charge of Tat was less than half that of the total negative charge of DZ01 (N/P = 1:0.34), the catalytic reaction was little affected. But, when the N/P ratio was further increased, the significant negative effect of Tat on DZ01 was observed, with the half-decreased *k*_obs_ at N/P = 0.68 and 1.36, and about one-fourth of the activity remained with N/P = 1:2.72. At the N/P ratio close to 1:5, a complete loss of activity occurred for DZ01. It was clear that the positively charged carrier had a concentration-dependent negative effect on the activity of 10-23 DNAzyme.

The capability of CPPs as delivery materials for oligonucleotide therapeutics has been extensively studied. It was suggested that consideration should be taken to keep a balance between the stability of the CPP-Oligonucleotide (ON) complex and the release of ON for its activity in cells [34]. The optimum N/P ratio of 2 was estimated for the Tat-ON complex formation [35]. Our present N/P ratio-*k*_obs_ relationship of 10-23 DNAzyme certified that the N/P ratio of 2 was also feasible for 10-23 DNAzyme, as the catalytic activity was still observed.

When DZ01 was mixed with lipofectamine 2000 (0.148 μL for 74 ng DZ01), according to the protocol, a half decrease in the activity was observed (Table 1). Based on the N/P ratio-dependent activity of the DZ01/Tat complex, the N/P ratio of DZ01 and lipofectamine 2000 was suggested to be around 1:1. Accordingly if less amount of lipofectamine 2000 was used, the negative effect on DNAzyme activity was decreased. With the suggested amount of lipofectamine 2000, efficient cleavage reactions have been observed in cell-based experiments [36,37,38].

These two positively charged carriers exerted a similar concentration-dependent effect on the behavior of 10-23 DNAzyme. Mg^2+^ was demonstrated to be still essential for the catalytic reaction. Therefore, we wondered whether their common electrostatic interaction with the negatively charged backbone of 10-23 DNAzyme might be responsible for the similar negative effect. Further investigations on the effect of Tat for the DNAzyme-substrate complex formation were conducted with CD spectra, thermal denaturation experiments, as well as non-denaturing PAGE analysis.

### 2.2. CD Spectra of DNAzyme-Substrate Complexes in the Presence of Tat

In the 10-23 DNAzyme-substrate complex, the recognition arm-substrate duplex formation and the base-stacking of the catalytic residues, as well as the electrostatic interactions with Mg^2+^, were suggested to be responsible for the active conformation. In the DNAzyme-Tat complex, the effect of the extra electrostatic interaction from the sidechains of Tat was studied on the DNAzyme-substrate (DZ01+D19) conformation, with wider N/P ratios (from 1:0.5 to 1:8). The antisense duplex D19+D19S was used to monitor the duplex formation in the presence of Tat with same N/P ratios.

**Figure 2:**
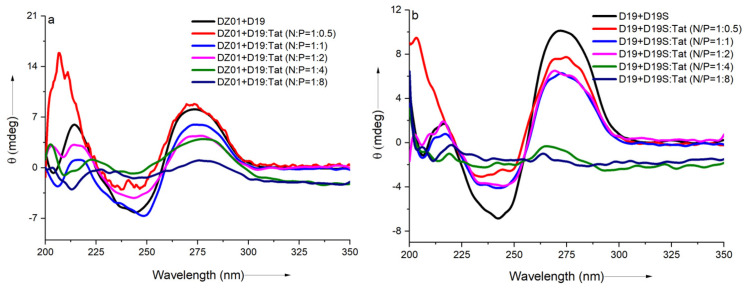
The effect of Tat on the CD spectra of the tertiary structures: (**a**) CD spectra of DZ01+D19 in the absence and presence of Tat at different N/P ratios; (**b**) CD spectra of D19+D19S in the absence and presence of Tat at different N/P ratios.

As shown in Figure 2a, a significant charge ratio-dependent effect was observed on the conformation of the DZ01+D19 complex. When the N/P ratio of DZ01/Tat was set at 1:0.5, the complex conformation was little disturbed by Tat; the majority of the DZ01 sequence could bind the substrate, resembling that of the free DZ01+D19 complex. At the same time, more positive charges (N/P ≥ 1:1) induced greater conformational changes. The characteristic duplex spectra disappeared gradually, especially at P/N = 4 or 8, because the higher N/P ratios led to more encapsulated oligonucleotides and less free DNAzyme for binding with the substrate. The large deviation of the tertiary structure induced by the extra cationic polymers could be responsible for the loss of the catalytic ability of the DNAzyme.

The regular duplex D19+D19S was used for comparison. As shown in Figure 2b, the duplex formation was also affected by the presence of Tat. The duplex conformation disappeared gradually with the increasing N/P ratios (from 1:0.5 to 1:8), which was in agreement with the conformational changes in the DZ01+D19 complex. Although the changes of the catalytic core could not be distinguished from the whole conformational changes, the decreased binding affinity of DNAzyme with the substrate was at least detrimental to the catalytic conformation and the reaction, which is in accordance with the negative effect of the mismatched pairing of the recognition arms of the DNAzymes [39]. On the other hand, the positively charged cargos were suggested to have a promoting effect on the product dissociation in the DNAzyme reaction; it is noteworthy that the binding of DZ01 with the substrate, as the first step toward the catalytic reaction, was also perturbed, leading to a negative effect on the DNAzyme activity.

### 2.3. Thermal Evaluation of DNAzyme-Substrate Complex in the Presence of CPP

**Figure 3:**
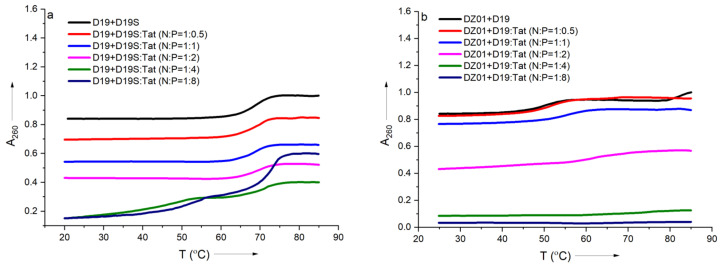
The effect of Tat on melting profiles of the tertiary structure formation: (**a**) the melting curve of DZ19+D19S in the absence and presence of Tat at different N/P ratios; (**b**) the melting curve of DZ01+D19 in the absence and presence of Tat at different N/P ratios.

In order to further confirm the effect of Tat on the substrate-binding ability of DZ01, the formation process of the complex DZ01+D19 and duplex D19+D19S were monitored by thermal annealing experiment. As shown in Figure 3a, the duplex D19+D19S formation was kept with extra Tat up to N/P = 1:2, and the same T_m_ (68 °C) as free D19+D19S was observed (Table S1). However, a further extra amount of Tat had a distinct destabilization effect. It was in accordance with the observations from CD spectra. DZ01+D19 complex had a lower T_m_ (51 °C) than the duplex D19+D19S since the former consists of two short duplex moieties spaced by a 15-mer catalytic loop, and there was a distinct transition between the folding state and the unfolding state. At the N/P ratios of 0.5:1 and 1:1, similar melting profiles to that of the free DZ01+D19 were observed (Figure 3b). When the N/P ratio was increased, the transition between the tertiary structure and single sequences became obscure, and the UV absorbance at A_260_ decreased accordingly, indicating that the catalytic conformation was changed and activity was negatively affected. Both CD spectra and T_m_ demonstrated that the DZ01/Tat complex with N/P ≤ 0.5 was appropriate for DZ01 in the base-pairing binding and the catalytic reaction, as large excess of Tat had a distinct destabilizing effect on the duplex formation.

### 2.4. PAGE Analysis of DZ01+D19 and D19+D19S Complexes in the Presence of Tat

**Figure 4:**
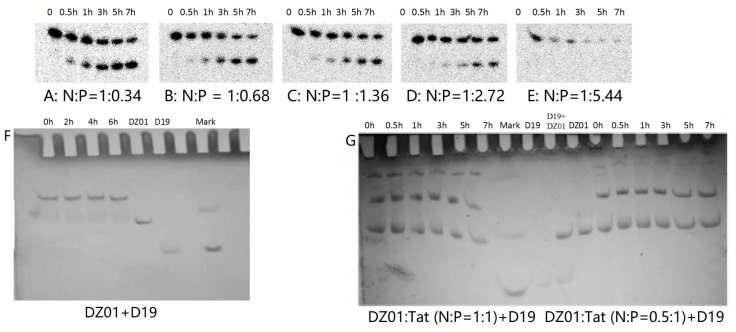

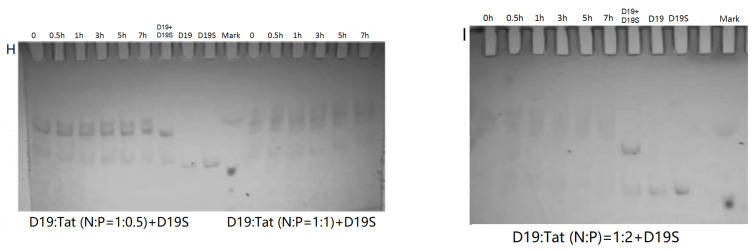
PAGE analysis of DZ01+D19 and D19+D19S in the presence of Tat. (**A**–**E**) denaturing PAGE of the catalytic reaction profiles of DZ01 in the presence of Tat with N/P of 1:0.34 (**A**), 1:0.68 (**B**), 1:1.36 (**C**), 1:2.72 (**D**), and 1:5.44 (**E**), with sampling time point: 0, 0.5, 1, 3, 5, 7 h. (**F**) DZ01+D19 complex formation in the reaction buffer. (**G**) DZ01/Tat (0.5:1 or 1:1) + D19 with different incubation times (0, 0.5, 1, 3, 5, 7 h); (**H**) D19/Tat (1:0.5 or 1:1) + D19S with different incubation times (1, 0.5, 1, 3, 5, 7 h); (**I**) D19/Tat (1:2) + D19S with different incubation times.

PAGE analysis was used to have insight into the influence of Tat on the reaction and complex formation of DZ01+D19. As shown in Figure 4, DZ01/Tat complexes with N/P from 1:0.34 to 1:2.72 could run the cleavage reaction (Figure 4A–D); the different cleavage percentages indicated that less free DZ01 was present with higher N/P ratios. Up to the N/P = 1:5.44 (Figure 4E), no cleavage reaction was observed for DZ01, and especially, longer incubation time led to more encapsulation of DZ01.

Next, the tertiary structure formation between DZ01 and D19, as well as the duplex formation between D19 and D19S, were then analyzed with non-denaturing PAGE. The tertiary structure DZ01+D19 could be formed and kept under present conditions (Figure 4F). In the presence of Tat with an N/P ratio of 1:0.5 and 1:1 (Figure 4G), the DZ01+D19 tertiary structure was still present; it was clear that DZ01 was disturbed from binding to D19. In the cases of greater N/P ratios (1:2, 1:4, and 1:8), no DZ01+D19 band could be observed because DZ01 was mostly encapsulated. Similarly, the duplex D19+D19S could be formed and kept under present conditions (Figure 4H,I). When D19 was pre-mixed with Tat of different N/P ratios (1:0.5, 1:1, and 1:2), the duplex formation was disturbed by the presence of extra Tat. From these results, the destabilizing effect of cationic polymers on tertiary nucleic acids is significant. Therefore, special attention needs to be paid to the charge ratio of cationic polymers when applying it in the biological evaluation of oligonucleotides.

## 3. Materials and Methods

### 3.1. Chemicals

The peptide Tat (GRKKRRQRRR) was synthesized on a microwave peptide synthesizer (Liberty, CEM Corporation, Mattews, NC, USA) with Fmoc protocol. After deprotection, the peptide was purified by HPLC on a preparative Kromasil^®^ C18 column (21.2 mm × 250 mm, 100 Å, 5 µm particle size, Sweden) using a gradient of 0–70% water in acetonitrile containing 0.1% TFA. And it was analyzed on a Shimadzu LC-10AT VP plus equipped with an SPD-10A VP Plus UV–Vis detector (at 210 nm) and a Kromasil^®^ C18 reverse-phase column (4.6 mm × 250 mm, 100 Å, 5 µm particle size, Sweden). Characterization was performed on a Bruker Reflex mass spectrometer (Bruker Daltonics, Inc., Billerica, MA, USA). Oligonucleotides DZ01, D19, and D19S were purchased from Qingke Biotech (Beijing, China), and the DNA-RNA-DNA substrate (D19R) from Takala (Dalian, China), and lipofectamine 2000 from Invitrogen (Life Technologies, Carlsbad, CA, USA). Other commercially available materials were used without further purification. In the case of Tat, the ratios of negative charges of DZ01 and positive charges of Tat (N/P) were calculated according to the molar concentration and net charges of each molecule. The amount of lipofectamine 2000 was used according to the protocol.

### 3.2. CD Spectra

DZ01 (3.37 μM, 0.5 mL) was mixed with Tat of different concentrations (6.74, 13.48, 26.96, 53.92, and 107.84 μM) in the reaction buffer (50 mM Tris-HCl, 4 mM Mg^2+^, pH 7.0) to reach the N/P of 1:05, 1:1, 1:2, 1:4, and 1:8. To the solution was added the D19 solution (3.37 μM, 0.5 mL, 50 mM Tris-HCl, pH 7.0), the DZ01-D19-Tat complexes stayed overnight for measurement, and the final concentration of Mg^2+^ was 2 mM.

D19 in the buffer (3.37 μM, 0.5 mL, 50 mM Tris-HCl, pH 7.0, 4 mM Mg^2+^) containing Tat of different concentrations (6.74, 13.48, 26.96, 53.92, and 107.84 μM) was mixed with D19S solution (3.37 μM, 0.5 mL, 50 mM Tris-HCl, pH 7.0), the D19-D19S-Tat complexes formed in the presence of 2 mM Mg^2+^. The samples were kept overnight before measurement. On a MOS-450 spectropolarimeter (Biologic, Claix, France), the CD spectra of each sample in a cuvette of 10 mm light path were measured at 20 °C. Three scans were averaged and smoothed with a Savitzky–Golay filter after subtraction of the background automatically.

### 3.3. T_m_ Measurement

The samples for CD spectra were used for thermal stability evaluation on a Varian Cary 3500 spectrometer. In a quartz cuvette of 10 mm light path, the sample was heated at 85 °C for 10 min; then, it was cooled at a rate of 1 °C/min, and the absorbance at 260 nm was recorded simultaneously. The melting curve was derivatized for the T_m_ value. At least two measurements were averaged, with a standard error of ±0.5 °C.

### 3.4. Nondenaturing PAGE Analysis of DZ01+D19 and D19+D19S in the Presence of Tat

D19 (2.3 OD) was dissolved in a buffer (90 μL, 2 mM Mg^2+^, 50 mM Tris-HCl). The Tat solutions of different concentrations were prepared with the same buffer. DZ01 (0.7 OD) was dissolved with the Tat solution (18 μL), and after incubation for 20 min at r.t., the D19 solution (18 μL) was added. The mixture was incubated at r.t., and samples (5 μL) were withdrawn at 0, 0.5, 1, 3, 5, and 7 h and mixed with glycerol (3 μL) for non-denaturing PAGE (20%) analysis. The control DZ01+D19 solution was prepared similarly; DZ01 was dissolved with the buffer instead of the Tat solution.

In the preparation of D19/Tat+D19S solution, D19S (3.5 OD) was dissolved in the buffer (90 μL, 2 mM Mg^2+^, 50 mM Tris-HCl). The Tat solution was prepared in the same buffer for different concentrations. D19 (0.7 OD) was dissolved in the Tat solution (18 μL), and the solution was incubated for 20 min at r.t. It was then mixed with D19S solution (18 μL) for the preparation of D19+D19S duplex. From the mixture, samples (5 μL) were withdrawn at 0, 0.5, 1, 3, 5, and 7 h and mixed with glycerol ((3 μL) for non-denaturing PAGE (20%) analysis.

### 3.5. Kinetics Measurement

DZ01-Tat complex or DZ01-lipofectamine 2000 complex was prepared by mixing DZ01 and Tat or lipofectamine 2000 of different concentrations in the buffer (50 mM Tris-HCl, pH 7.0, 4 mM Mg^2+^).

The substrate D19R was radioactively labeled with [γ-^32^P]ATP with TK4 polymerase at 37 °C for 40 min. The reaction was stopped by heating at 70 °C for 10 min. After cooling to r.t., the solution was absorbed on a Sep-PAK column and washed with water. The product was eluted with methanol/H_2_O (70/30, *v*/*v*) and evaporated in a vacuum. It was then dissolved in water, and the UV absorbance at 260 nm was measured for quantification with a molar extinction coefficient of DZ01 (ε 29,600).

To the radio-labeled D19R solution in the buffer (25 nM, 50 mM Tris-HCl, pH 7.0) was added the encapsulated DZ01 complex to start the reaction. In the reaction system, DZ01 (148 nM) and D19R (1.48 nM) were used for the evaluation; in the presence of 2 mM Mg^2+^, the amount of Tat peptide was calculated according to the N/P ratios, and lipofectamine (0.148 μL) was used according to the Protocol. The effect of lipofectamine was studied further with decreased amounts of lipofectamine (0.074 μL and 0.037 μL). Samples were withdrawn from the reaction mixture and mixed with an equal volume of the stopping solution (8 M urea, 0.1 M EDTA) at certain time points. The samples from one reaction were analyzed with denaturing PAGE (20%, 8 M urea) and imaged with a PhosphoImager (Cyclone Plus Phosphor Scanning System, PerkinElmer, Waltham, MA, USA). The observed rate constants were calculated with the equation P % = P_∞_% − C exp [−*k*_obs_t], where P is the percentage of the cleaved product at time t, C is the difference of P% between t = ∞ and t = 0, and P_∞_ is the endpoint, an endpoint of 90% product was assumed. The data were then averaged with results of at least three independent experiments with a variation of less than 20%.

## 4. Conclusions

In conclusion, in the Mg^2+^-mediated catalytic cleavage reaction of 10-23 DNAzyme, the presence of the positively charged Tat or lipofectamine 2000 had a negative effect on the catalytic activity. With the thermal transition, CD spectra, and PAGE analysis, it was demonstrated that the electrostatic interaction was an unfavorable factor in the design of delivery materials, as the random electrostatic interaction could deter from forming the single active DNAzyme-substrate complex and the duplex structure, which is the first critical step for all the antisense-based oligonucleotide therapeutics (antisense, ribozymes, DNAzymes, siRNA, miRNA, anti-miRNAs, as well as CRISPR). Therefore, CPPs and lipofectamines are being improved for new generations, and ionizable and neutral lipid nanoparticles and ligand conjugates are being developed as choices for the delivery of therapeutic oligonucleotides.

## Figures and Tables

**Table 1:** The observed rate constants of DNAzyme-polymer complexes with different N/P ratios under the single turnover conditions in the presence of 2 mM Mg^2+ 1^.

DZ01/Tat	*k*_obs_ (min^−1^)	DZ01/Lipofectamine	*k*_obs_ (min^−1^)
DZ01	0.0051 ± 0.0007		
N/P = 1:0.34	0.0044 ± 0.0001	1:0.25	0.0046 ± 0.0001
N/P = 1:0.68	0.0025 ± 0.00001	1:0.5	0.0036 ± 0.0005
N/P = 1:1.36	0.0024 ± 0.00006	1:1	0.0022 ± 0.0001
N/P = 1:2.72	0.0011 ± 0.0001		
N/P = 1:5.44	- ^2^		

^1^ Conditions for the cleavage reaction: DZ01 (2.5 μM) encapsulated with positive polymers of different N/P ratios on D19R (25 nM), 2.0 mM Mg^2+^ was added to start the cleavage reaction at 37 °C. ^2^ *k*_obs_ < 0.0001.

## Data Availability

The data presented in this study are available in this article.

## Supplementary Materials

The following supporting information can be downloaded at: https://www.mdpi.com/article/10.3390/molecules28093942/s1, Table S1: Thermal evaluation results of the complex DZ01-D19 and the duplex D19-D19S in the presence of Tat.

## Sample Availability

Samples of the compounds are available from the authors.
