# Supplementary material for: Studies on the Effect of Lipofectamine and Cell-Penetrating Peptide on the Properties of 10-23 DNAzyme
Source: Molecules. 2023 May 7;28(9):3942. doi: 10.3390/molecules28093942 (PMC10179765; doi:10.3390/molecules28093942)
Supplement: Supplementary file 1 [file molecules-2312977-supplementary.pdf › molecules-2312977-supplementary.pdf]

Supplementary materials

Studies on the Effect of Lipofectamine and Cell-Penetrating Peptide on the Properties of 10-23 DNAzyme

Huanhuan Liu, Yang Li, Shanshan Du, Chenhong Wang, Yuexiang Li, Ruiyuan Cao, Weiguo Shi, Shihui Liu and Junlin He

Table S1 Thermal evaluation results of the complex **DZ01-D19** and the duplex **D19-D19S** in the presence of CPP[a]

**DZ01-D19**/CPP	Tm (°C)	**D19-D19S**/CPP	Tm (°C)
**DZ01-D19**	52	**D19-D19S**	68
**DZ01**+ CPP (N/P = 1:05)	51	**D19**+ CPP (N/P = 1:05)	68
**DZ01**+ CPP (N/P = 1:1)	52	**D19**+ CPP (N/P = 1:1)	68
**DZ01**+ CPP (N/P = 1:2)	-	**D19**+ CPP (N/P = 1:2)	68
**DZ01**+ CPP (N/P = 1:4)	-	**D19**+ CPP (N/P = 1:4)	-
**DZ01**+ CPP (N/P = 1:8)	-	**D19**+ CPP (N/P = 1:8)	-

[a] ± 0.5 °C (2 mM Mg^{2+}) for T_m measurement.
